# Therapeutic Potential of *Dioscorea* Extract (DA-9801) in Comparison with Alpha Lipoic Acid on the Peripheral Nerves in Experimental Diabetes

**DOI:** 10.1155/2013/631218

**Published:** 2013-03-12

**Authors:** Heung Yong Jin, Sun Hee Kim, Hea Min Yu, Hong Sun Baek, Tae Sun Park

**Affiliations:** ^1^Division of Endocrinology and Metabolism, Department of Internal Medicine, Research Institute of Clinical Medicine of Chonbuk National University, Biomedical Research Institute of Chonbuk National University Hospital, Chonbuk National University Medical School, 634-18, Keum-Am Dong, Jeonju 561-712, Republic of Korea; ^2^Division of Endocrinology and Metabolism, Department of Internal Medicine, Research Institute of Clinical Medicine, Eulji University Hospital-Eulji University, Daejeon, Republic of Korea

## Abstract

DA-9801, a mixture of extracts from *Dioscorea japonica* Thunb. and *Dioscorea nipponica* Makino, was reported to have neurotrophic activity. Therefore, we investigated the therapeutic potential of DA-9801, in comparison with alpha lipoic acid (ALA), for peripheral nerves preservation in experimental diabetes. Experimental animals were divided into 4 groups, and each group was designated according to the type of treatment administered as follows: normal, DM, DM+DA-9801, and DM+ALA. After 16 weeks, response thresholds to tactile and thermal stimuli were higher in DM+DA-9801 group than in nontreated DM group. This degree of increase in DM+DA-9801 group indicates more therapeutic potency of DA-9801 than ALA. Western blot analysis showed more significant increase in NGF and decrease in TNF-*α* and IL-6 in DM+DA-9801 group than in DM or DM+ALA groups (*P* < 0.05). IENF density was reduced less significantly in the DM+DA-9801 group than in other DM groups (7.61 ± 0.32, 4.2 ± 0.26, and 6.5 ± 0.30 in DM+DA-9801, DM, and DM+ALA, resp., *P* < 0.05). Mean myelinated axonal area in the sciatic nerves was significantly greater in DM+DA-9801 group than in other DM groups (69.2 ± 5.76, 54.0 ± 6.32, and 63.1 ± 5.41 in DM+DA-9801, DM, and DM+ALA, resp., *P* < 0.05). Results of this study demonstrated potential therapeutic applications of DA-9801 for the treatment of diabetic peripheral neuropathy.

## 1. Introduction

Half of diabetic patients have chronic complication involving the peripheral nervous system, which results in diabetic peripheral neuropathy (DPN). Besides morbidity and mortality, DPN has diverse symptoms, which leads to the deterioration of the quality of life of diabetic patients [1, 2]. DPN can be managed via pathogenic correction and symptomatic control [3]. Alpha lipoic acid (ALA), gamma linoleic acid, neurotrophic agent, and aldose reductase inhibitor are well known as pathogenic treatments based on multifactorial DPN mediators [3, 4]. Among these, ALA is widely used for the clinical pathogenic treatment of DPN. On the contrary, the therapeutic effectiveness of neurotrophic agents, such as nerve growth factor (NGF), for DPN treatment is not yet confirmed because clinical trials could not yield positive results, although neuronal effects were claimed by several experimental studies [5, 6]. However, defective neurotrophic factors binding to specific receptors have been suggested as one cause of DPN, and preventing this mechanism has been regarded as a possible therapy for DPN. 

DA-9801 is a mixture of extracts from *Dioscorea japonica* known as SanYak and *Dioscorea nipponica* known as Buchema [7]. Results of previous studies on DA-9801 showed increased NGF level and improvement of nociceptive pain, although the exact role of NGF and the degree of its neurotrophic effect compared to other agents were not described [8]. In this context, DA-9801, an enhancer of NGF, can then be postulated to play a role in the pathogenic treatment of DPN similar to other neurotrophic factors. Therefore, in this study, we investigated the neuroprotective potential of DA-9801 on the peripheral nerves of streptozotocin- (STZ-) induced diabetic rats, in comparison with ALA.

## 2. Materials and Methods

### 2.1. Animals, Materials, and Experimental Design

Six to eight-week-old male Sprague-Dawley (SD) rats weighing 160 to 180 g were purchased from Damool Science (Daejeon, Chungnam, Korea). Animals were housed in an optimal condition with a 12 hr light and dark cycle. The room temperature was maintained at 23 ± 1°C and humidity at 53 ± 2%. The animals had a free access to food and water. All experiments and protocols were performed after the approval by the Institutional Rat Care and Use Committee of the Chonbuk National University Medical School (CBU 2011-0055). Streptozotocin (STZ) (Sigma Chemical, St. Louis, MO, USA) dissolved in 0.1 mol/L sodium citrate buffer (pH 4.5) was injected intraperitoneally to experimental rats (60 mg/kg body weight) to induce diabetes. Forty-eight hours after STZ injection, rats showing blood glucose levels higher than 350 mg/dL were verified to have diabetes. After subjecting rats into an overnight fasting, blood samples were drawn from the tail vein, and blood glucose levels were measured using Precision Xtra Plus (Abbott Laboratories, MediSence Products, Bedford, MA, USA). In the same manner, age-matched control rats received an equal volume of the vehicle-sodium citrate buffer (pH 4.5). DA-9801 was supplied from Dong-A Pharmaceutical (Yongin, South Korea), while alpha lipoic acid (ALA) was supplied from Bukwang Pharmaceutical (Seoul, South Korea). After 4 weeks of STZ and sodium citrate buffer injection, normal and diabetic rats were randomly selected and divided into 4 groups (*n* = 10 per group) according to the treatment agents: normal (normal control group), DM (nontreated diabetic group), DM+DA-9801 (DA-9801-treated diabetic group), and DM+ALA (ALA-treated diabetic group). DA-9801 was administered orally at a dose of 100 mg/kg/day, while 0.5% ALA, which was in powdered form, was mixed with daily food at a dose of 50 mg/kg/day. On the 8th and 16th weeks of treatment, behavioral assessment and morphometric comparison were performed. On the 17th week, biochemical parameters including NGF, TNF-*α*, and IL-6 levels in sciatic nerve and spinal cord were measured.

### 2.2. Behavioral Assessment by Tactile Response and Thermal Response

To measure the allodynia, tactile stimulation was performed using flexible von Frey filament (Stoelting Co., Illinois, USA) to evaluate the withdrawal threshold of hind paw. After adaptation to the testing condition for at least 20 min, rats were placed individually in a plastic cage with 1 cm sized perforated mesh. Von Frey filament, with calibrated bending forces (in g), were applied perpendicularly to the plantar surface of the hind paw to deliver tactile stimuli of varying intensity. This test was conducted according to the procedure of Chaplan et al. [9]. The stimulation was performed five times with 5-sec and interval, and immediate withdrawal in at least one time in the five-time application was determined to be a positive response. To measure thermal response, rats were placed on a hot plate (Ugo Basile, PA, USA) with temperature at 55 ± 1°C. The latency to the first sign of paw licking response to avoid the heat was taken as a threshold for heat sense.

### 2.3. NGF, TNF-*α*, and IL-6 Determinations in Sciatic Nerve and Spinal Cord

On the 17th week, sciatic nerves and spinal cord were rapidly removed after killing all rats under deep anesthesia and were prepared for western blot analysis. Sciatic nerve and spinal cord were broken down mechanically with 100–200 *µ*L of Triton lysis buffer. After centrifugation at 13,200 rpm for 15 min at 4°C, the supernatant was transferred to a fresh tube (1.5 mL capacity), and the protein concentration was quantified following Bradford method [10]. Samples were loaded into 10% SDS polyacrylamide gel, and blocking of nonspecific binding was achieved by placing the membrane in a dilute protein solution of nonfat dry milk (5% skim milk) with 1% Tween-20 in PBS for 3 hr at room temperature. The blots were incubated with primary antibodies such as NGF, TNF-*α*, and IL-6 (1 : 1,000; Abcam, UK) overnight at 4°C. The membranes were washed in 1 TBST by shaking 3 times for 10 min. Incubation with HRP-conjugated secondary antibody donkey anti-goat IgG, HRP conjugate (1 : 2,000; Santa Cruz Biotechnology, Santa Cruz, CA, USA) was performed on a rocker for 1 hr at 4°C. Protein bands were detected using ECL-plus kit (Amersham Pharmacia Biotech, Buckinghamshire, England). Quantitative image analysis was conducted using LAS 3000 Fuji film and film densitometry was performed using MultiGauge version 3.0 (Fuji film).

### 2.4. Morphometric Assessment

On the 0th and 8th weeks, 3 × 3 mm tissues were taken from the dorsum of the foot via skin biopsy for immunohistochemical analyses of intraepidermal nerve fiber (IENF). On the 17th week after killing all rats under deep anesthesia, last cutaneous tissue samples from the feet and segments of the sciatic nerve were obtained from each rat. Sciatic nerve tissue samples were immersed in a fixative (2.5% glutaraldehyde in phosphate-buffered saline (PBS)) and incubated overnight at 4°C. These samples were then embedded in JB-4 solution (Polysciences, Inc., Germany), and 1.5 *μ*m transverse sections were stained with toluidine blue. The procedures used for immunohistochemical analysis were the same as those described previously [11]. Skin tissue specimens were fixed with periodate-lysine-paraformaldehyde (PLP) (2% paraformaldehyde, 0.075 M lysine, 0.05 M phosphate buffer pH 7.4, 0.01 M sodium m-periodate) solution for 24 hr. After thorough rinsing in PBS containing 20% glycerol-0.1 M phosphate buffer for 48 hr at 4°C, the tissue specimens were cryoprotected with Tissue-Tec (OCT compound) (Miles, Elkhart, IN, USA). Sections of 40 *μ*m in thickness, cut perpendicular to the dermis, were prepared with a sliding cryostat (Leica CM 1510, Leica Microsystems AG, Wetzlar, Germany) and were immersed in PBS for 15 min at room temperature. Samples were then transferred into microtubes containing Dako Protein Block Serum Free (Dako, Carpinteria, CA, USA) as a blocking buffer supplemented with 3% goat serum. After 30 min of blocking on a shaker table at room temperature, specimen sections were washed with PBS twice for 10 min and then incubated overnight with primary antibody, rabbit anti-protein-gene-product 9.5 (PGP 9.5) (Biogenesis, Poole, UK) at a dilution of 1 : 100 at 4°C. The antibodies were diluted in antibody diluent (Dako, Carpinteria, CA, USA) supplemented with 1% goat serum. After complete washing, the specimens were incubated with the secondary antibody, goat anti-rabbit IgG-FITC (1 : 200, Vector, UK), for 1 hr at room temperature in a dark room. After washing with PBS, sections were placed on slides and mounted with a fluorescent mounting media (Dako, Carpinteria, CA, USA).

Photomicrographs of the myelinated fiber and IENF were captured using a digital camera (Axiocam HRC, Carl Zeiss, Goettingen, Germany) with a final magnification of 400 and 100 times, respectively. The myelinated fiber or axonal area in the sciatic nerve, represented by the outer or inner border of the myelin sheath, was measured with the aid of analySIS image software (Soft Imaging Systems GmbH, Munster, Germany), and the mean myelinated fiber area was determined. In addition, the thickness of myelin sheath and the diameter of axonal fiber were measured.

PGP 9.5-immunoreactive nerve fibers in the epidermis of each section were counted as described previously [12]. In cutaneous nerves, each nerve fiber with branching points inside the epidermis was counted as one fiber. The number of intraepidermal nerve fibers (IENFs) per length (fibers/mm) was considered as the amount of innervation. In order to avoid any possible bias during preparation and calculation, two independent investigators were blinded to the experimental groups. Moreover, the slides were mixed with a set of normal slides before examination.

### 2.5. Statistical Analysis

All data were expressed as means ± SD. One-way ANOVA with Duncan's post hoc test was used to compare experimental groups. The confidence interval for testing the differences was 95%, and analyses results with *P* < 0.05 were considered as statistically significant. Statistical analyses were performed using SPSS 12.0 software (SPSS Inc., Chicago, IL, USA).

## 3. Results

### 3.1. Effect of DA-9801 on the Body Weight and Blood Glucose Level

**Figure 1:**
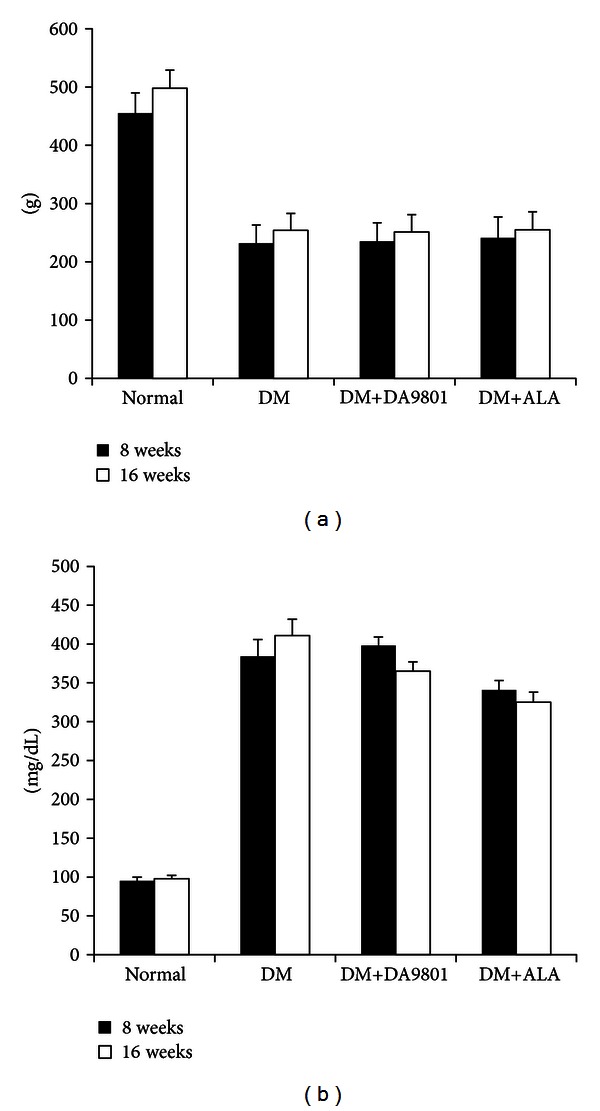
Body weight (a) and blood glucose levels (b) of the experimental groups. Body weight was increased gradually in normal glucose rats. In the diabetic groups, however, it was not increased irrespective of DA-9801 or ALA treatment. DA-9801 and ALA had no effects on blood glucose levels. Data are presented as means ± SD (*N* = 10 in each group). ALA: Alpha lipoic acid and DM: diabetes.

Two weeks after STZ injection, increased blood glucose levels and decreased body weight were observed in diabetic groups compared with normal rats. Treatment with DA-9801 for 16 weeks did not affect on the body weight and blood glucose levels in DM+DA-9801 group. There were no significant changes in body weight and blood glucose levels in the DM+ALA group (Figures 1(a) and 1(b)).

### 3.2. Effect of DA-9801 on the Tactile Response and Thermal Response

**Figure 2:**
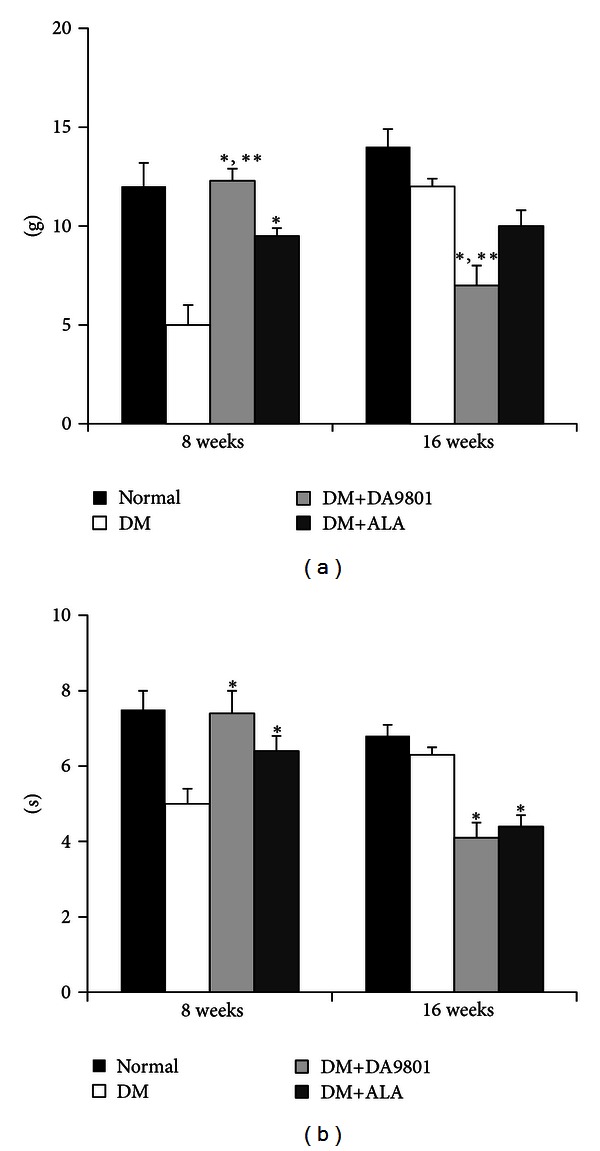
Von Frey filament (a) and hot plate (b) response of the experimental groups. Threshold of tactile response was lower in the nontreated diabetic group as compared with the DM+DA-9801 or DM+ALA groups on the 8th week. On the 16th week, however, this trend was reversed, and the thermal response was also changed in a similar pattern to the tactile response. Data are presented as means ± SD. *P < 0.05 versus Normal and **P < 0.05 versus DM (*N* = 10 in each group). ALA: Alpha lipoic acid and DM: diabetes.

On the 8th week, the paw withdrawal threshold, when stimulated with von Frey filament, was reduced by 59% in the diabetic group compared with normal group. However, this reduction was significantly prevented in DM+DA-9801 and DM+ALA groups compared with nontreated diabetic group (Figure 2(a)). The degree of preventing threshold reduction was observed more potently in DM+DA-9801 than in DM+ALA group. On the 16th week, this pattern of response to von Frey filaments was reversed in the DM+DA-9801 or DM+ALA groups, and more blunted response was observed in the nontreated diabetic group. However, DM+DA-9801 group exhibited significantly preserved sensitivity compared with other diabetic groups (*P* < 0.05) (Figure 2(a)). On the 8th week, the latent time to withdrawal of rat paw on the hot plate was significantly reduced (by 34%) in diabetic group compared with normal group. DA-9801 treatment prevented this hypersensitive response in diabetic group, and the latent time to withdrawal of rat paw was longer in DM+DA-9810 group than in DM+ALA group, although the difference was not significant. However, this pattern was reversed in the diabetic groups after 16 weeks, DM+DA-9801 and DM+ALA groups showed more preserved sensitivity to hot stimulus compared with the nontreated diabetic group (Figure 2(b)). 

### 3.3. Effect of DA-9801 on the NGF, TNF-*α*, and IL-6 Levels in Sciatic Nerve and Spinal Cord

**Figure 3:**
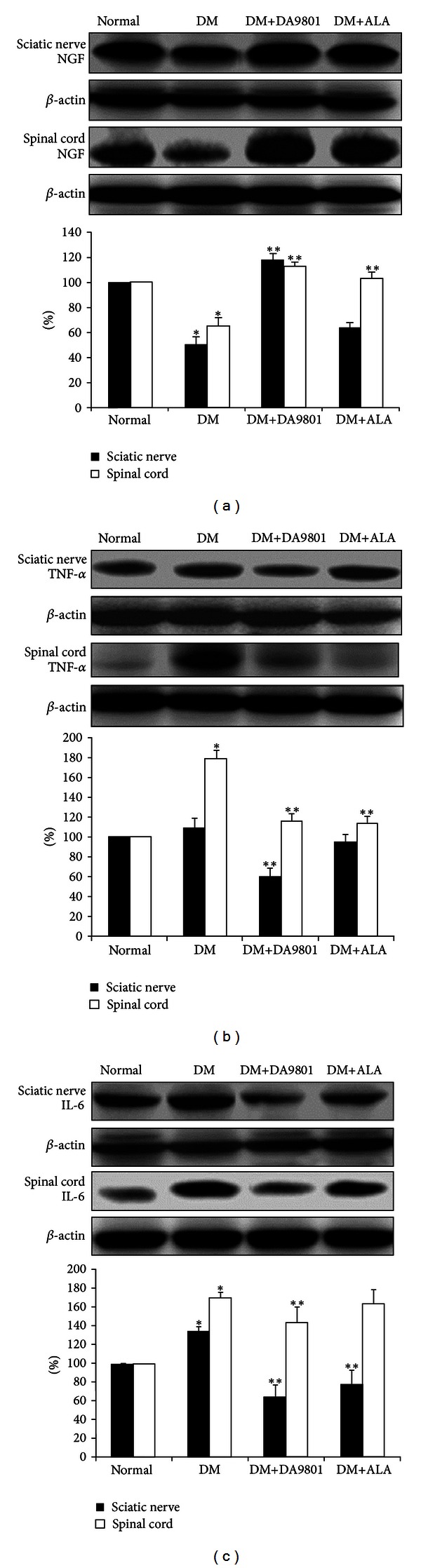
NGF, TNF-*α*, and IL-6 levels in the sciatic nerve and spinal cord of the experimental groups. Data are presented as means ± SD. *P < 0.05 versus Normal and **P < 0.05 versus DM (*N* = 10 in each group). ALA: Alpha lipoic acid and DM: diabetes.

NGF level in sciatic nerve and spinal cord was markedly decreased (about 50%) in the non-treated diabetic group compared with those in normal ones. However DM+DA-9801 group recovered NGF level to a similar degree with the normal group. This degree of NGF recovery was more potent in DM+DA-9801 than in DM + ALA group, although ALA is not an NGF inducer (Figure 3(a)). TNF-*α* and IL-6 levels increased in diabetic condition groups compared with the normal ones. These cytokines in each diabetic group were reduced after DA-9801 treatment, and the degree of reduction was similar in DM+DA-9801 and DM+ALA groups (Figures 3(b) and 3(c)).

### 3.4. Morphometric and Quantitative Comparisons of Peripheral Nerve among Groups

**Figure 4:**
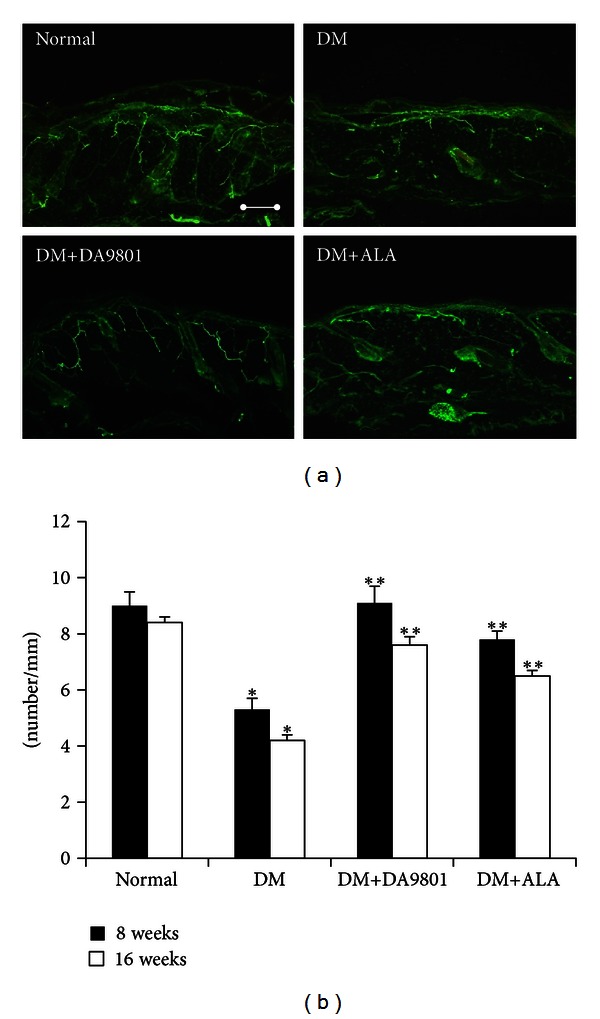
Immunohistochemistry of cutaneous small nerve fibers of the foot dorsum (a) and the IENF density (b) of the experimental groups. PGP 9.5-stained small nerve fibers are more preserved and less shortened, and IENF density was higher in the DM+DA-9801 group than its non-DA-9801-treated DM groups. Data are presented as means ± SD. *P < 0.05 versus Normal and **P < 0.05 versus DM (*N* = 10 in each group). ALA: Alpha lipoic acid and DM: diabetes. Bar indicates the 100 *µ*m.

**Figure 5:**
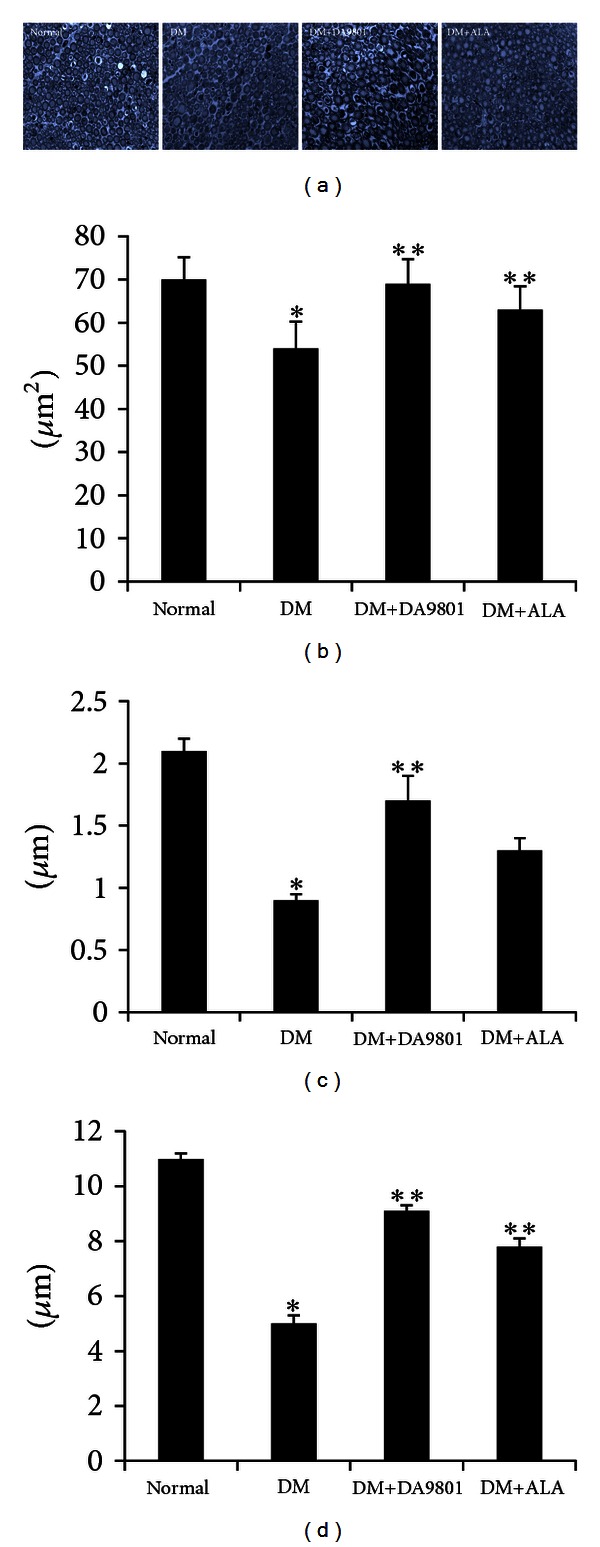
Immunohistochemistry of the sciatic nerve (a) and the area of myelinated nerve fiber (b), the diameter of myelin sheath (c), and the diameter of axon (d) of the experimental groups. Myelinated axonal nerve fibers were less degenerated in the DM+DA-9801 and DM+ALA groups. In addition, the size of nerve fibers with or without myelin sheath was increased in the DM+DA-9801 and DM+ALA groups as compared with the DA-9801-treated DM groups. Data are presented as means ± SD. *P < 0.05 versus Normal and **P < 0.05 versus DM (*N* = 10 in each group). ALA: Alpha lipoic acid and DM: diabetes.

The comparison of cutaneous peripheral nerve quantity showed markedly reduced IENF density in non-treated diabetic group by about 42–50% compared with normal group. Shortened and degenerated patterns of small peripheral nerve fibers were observed in diabetic groups (Figure 4(a)). However, this morphological change and the reduction of IENF density were blunted in DM+DA-9801 and DM+ALA groups. Furthermore, DA-9801 showed more potent therapeutic potential than ALA, although this difference did not reach statistical significance (Figures 4(a) and 4(b)). The examination of the sciatic nerves also showed similar trend of results on IENF density. Mean area of myelinated axonal fiber was reduced in non-treated diabetic groups compared with the normal ones. This reduction was prevented in the DM+DA-9801 group, which shows more potent therapeutic potential of DA-9801 than ALA. The diameter of myelin sheath and the axonal fiber of DM+DA-9801 or DM+ALA groups increased significantly compared with the non-treated diabetic group (Figure 5).

## 4. Discussion

Peripheral neuropathy is a common disease in diabetic patients, and an estimate of more than half of all diabetic patients suffer from diverse neuropathic symptoms [13]. Diabetic peripheral neuropathy (DPN) occurs as a result of multiple etiologies and leads to neuronal damage. Affected lower leg (e.g., diabetic foot) may lead to ulcer or amputation, which lowers the quality of life of the patients [14]. The basic pathogenic mechanism of DPN can be divided into metabolic and vascular etiologies. A number of mechanisms suggested to be a pathogenesis of DPN have been investigated, which include polyol pathway, advanced glycation end product, and activation of the proteins kinase, poly ADP-ribose polymerase, and aldose reductase [15, 16]. These can result in increased oxidative stress and attenuation of antioxidative defense mechanism. Therefore, hyperglycemia, a metabolic disruption causing increased oxidative stress in the peripheral nerves, is important to the pathogenesis of DPN [17]. In patients with defective antioxidant response, oxidative stress from hyperglycemia can cause vascular impairment leading to endoneurial hypoxia [18]. In addition to these etiologies, diminished nerve growth factor also causes problems in neuronal survival, growth, and differentiation, although the extent of its contribution to the pathogenesis of DPN is unclear [5, 19]. Previous studies suggested that NGF plays a significant role in the pathogenesis of DPN, and NGF or NGF mimetics administration may recover the neuronal damage caused by DPN, although no successful clinical trials to show the efficacy of NGF were reported [5, 20, 21]. Thus, neuroprotective effect of various neurotrophic or NGF mimetic agents on diabetes had been tested [21, 22].

Hence, in the present study, we investigated the potential of a botanical agent (DA-9801) in increasing NGF levels for the treatment of DPN. A comparative study with ALA, which is a well-known agent for the treatment of DPN, was also conducted. 

Various species of Dioscorea has been used as a traditional medicine for diverse diseases including metabolic disorders, inflammatory diseases, pain control, and neuropathic diseases [23–25]. DA-9801 is a mixture of extracts from Dioscorea japonica Thunb (DJ) and Dioscorea nipponica Makino (DN). Previous studies showed neurotrophic activity of DA-9801 *in vitro* and in experimental animals [7, 8]. However, there are few data to show the degree and the scientific mechanism of the neuroprotective effect of Dioscorea species on diabetic animals. In this study, we assessed the functional parameters, related mediators, and morphometric and quantitative characteristics of DPN in STZ-induced diabetic rats after 16 weeks of DA-9801 treatment. In this study, DA-9801 did not show significant effect on the body weight and blood glucose levels of experimental rats. However, deteriorating sensitivities caused by hyperglycemia were blunted through DA-9801 treatment. The degree of this response was more potent in DM+DA-9801 group than in the DM+ALA group. Histologically, DPN is characterized by axonal degeneration, demyelination, and neuronal atrophy associated with neuronal regeneration failure [26]. These neuronal damage from hyperglycemia leads to diverse neuronal symptom and lower leg problems in diabetic patients. Clinically and experimentally, the assessment of neuropathic symptoms is not reliable as an absolute indicator for the diagnosis or the assessment of the severity of peripheral nerve damage in diabetic patients. Furthermore, DPN can be manifested by positive or negative sensory symptoms, with or without sensory motor deficit. Mixed symptoms of DPN are also possible depending on the nerve damage, which is related to glycemic control and diabetes duration. Therefore, subjective and objective assessment should be incorporated in the diagnosis and assessment of the degree of DPN. Therefore, our data showing response threshold to mechanical and thermal stimuli need to be interpreted in association with the quantity of peripheral nerve. On the 8th week, more sensitive reaction to Von Frey filament and hot plate was observed in the diabetic group than in normal group. However, this sensitivity was reduced through DA-9801 and ALA treatments. This trend was more pronounced in DM+DA-9801 group than in DM+ALA group, although this difference was insignificant. Results also showed decreased IENF density in diabetic rats, which suggests that more hypersensitive reaction may result from degeneration of small nerve fiber in non-treated diabetic group. On the 16th week, the sensitive response observed on the 8th week was reduced in diabetic group compared with the normal ones. However, treatment with DA-9801 and ALA has blunted this insensitivity, in agreement with the results on IENF. These results suggest that DPN progression may be prevented by DA-9801 or ALA treatment, which may also reverse the pattern of mechanical allodynia and thermal response in the 16th week compared with the results in the 8th week in diabetic rats. However, the comparison and interpretation of functional parameters at the later stages of diabetes are difficult because of possible mixed symptoms and sensitivity. Furthermore, body weight difference between normal and diabetic groups may result in different mechanical allodynia and thermal response thresholds. Thus, functional parameters including tactile and thermal responses between normal and diabetic groups in this study need to be compared in consideration with body weight differences, although ANCOVA was used to exclude such bias. These types of behavioral data also require careful interpretation as sick animals, such as those with long-term diabetes, can become unresponsive to sensory stimuli due to generalized cachexia rather than something related to neuronal degeneration or dysfunction.

In the body weight and glucose levels, DA-9801 did not show the lowering effect in diabetic group. Of course, DA-9801 is not lowering agent for glucose or body weight, and our animal model was late state type 2 diabetic or type 1 diabetic model using STZ 60 mg/kg. Therefore, we thought that only one oral agent is difficult to lower the blood glucose in this condition, even though DA-9801 may have the glucose lowering effect. Accordingly, we thought that body weight was difficult to be affected without glucose control in this animal model because this agent could not affect on the food intake.

**Figure 6:**
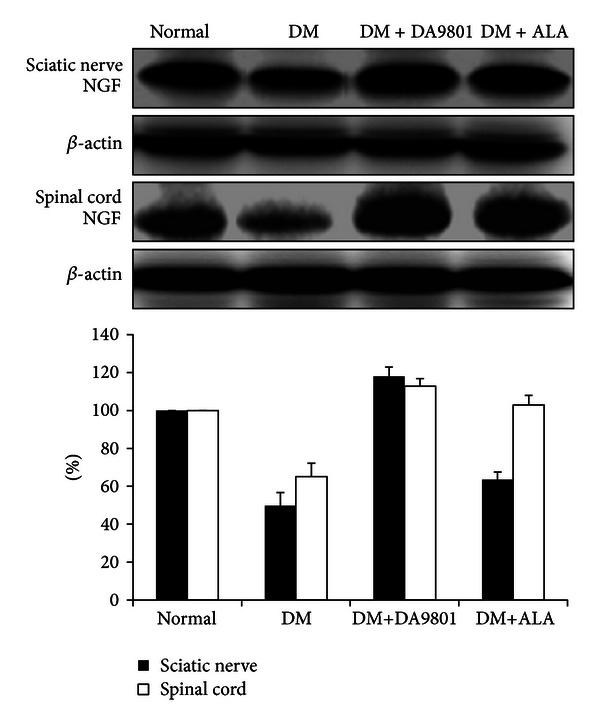


**Figure 7:**
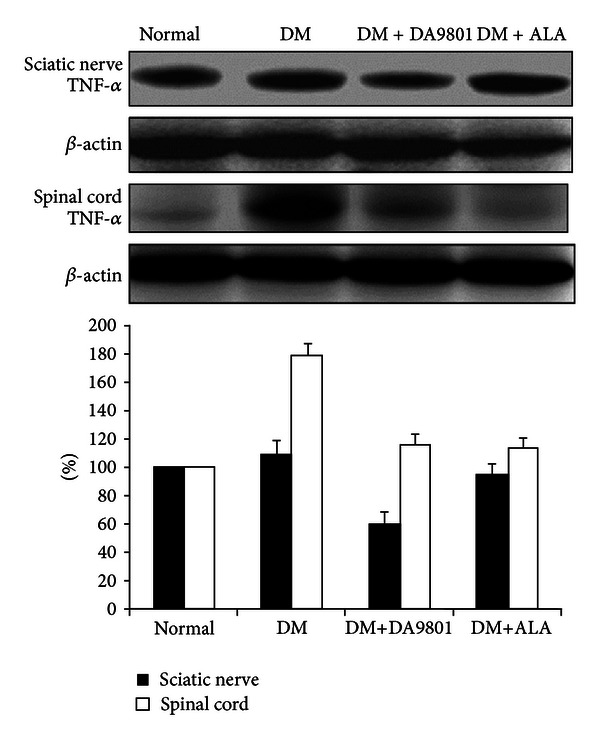


**Figure 8:**
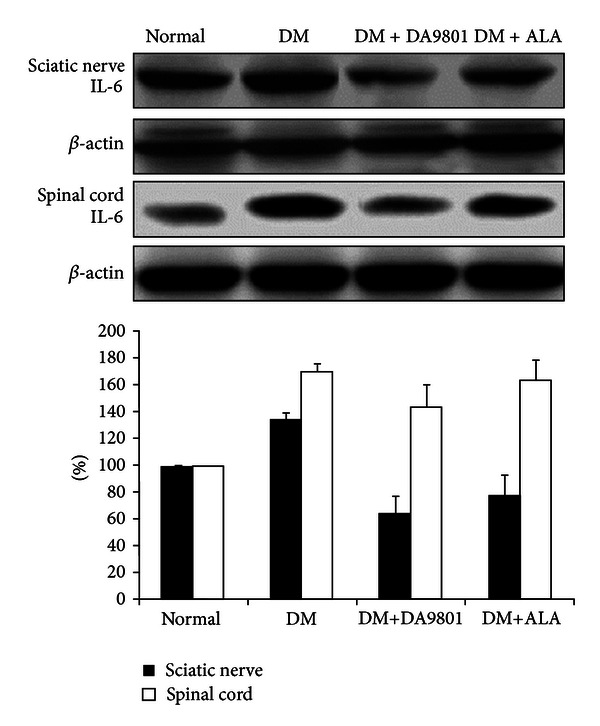


Anti-inflammation, antioxidant, or neuronal regeneration are fundamental mechanisms for nerve protection, although more detailed diverse pathways including several mediators are involved in DPN pathogenesis [27, 28]. These combined pathogenic corrections targeting neurotrophic and anti-inflammation can give more potent benefit than single pathogenic modulation of oxidative stress. The detailed mechanism of NGF in neuronal protection is known to phosphorylate Trk-A and induce PI3K pathways, although direct NGF administration did not reach the nervous tissue [21, 29–31]. Hence, neurotrophin-enhancing agents are more interesting in this respect. To investigate the neuroprotection mechanism of DA-9801 in diabetic rats, we examined the NGF concentration in the sciatic nerve and spinal cord (Figures 6, 7, and 8). However, complete prevention of DPN by single factor correction is difficult due to the complexity and diversity of etiologies in the pathogenesis. Hence, we measured other mediators, such as TNF-*α* and IL-6, which are well known to be involved in DPN pathogenesis. In the present study, the increases of TNF-*α* and IL-6 in diabetic group were reduced after DA-9801 treatment. Therefore, neuronal effect mediated by increased NGF level and anti-inflammation mediated via TNF-*α* and IL-6 may have played a role in the peripheral nerve protection in DM+DA-9801 group. However, the degree of the contribution of NGF and cytokines on DPN treatment has not been confirmed.

To support our claim of therapeutic potential of DA-9801 for peripheral nerve protection, the structural change of cutaneous small nerve fibers and sciatic nerves was also compared among experimental groups. We found that the quantity of small intraepidermal nerve fibers was more preserved, and the pattern was less degenerated in DM+DA-9801 and DM+ALA groups than in non-treated diabetic group. Moreover, the degree of neuronal protection of DA-9801 was found to be more potent than ALA. Based on IENF density measurements, DM+DA-9801 or DM+ALA groups exhibited less degenerated morphology of myelin sheath and axon compared with the non-treated diabetic group. DA-9801 also showed more potent therapeutic potential than ALA in the present study. Taking all results as a whole, this study showed that DA-9801 can act as a biochemical and morphological protective agent on the peripheral nerves in diabetic patients. However, the potential of DA-9801 to induce other neurotrophic factors or improve other mediators involved in pathogenesis of DPN remains to be determined. Aside from NGF, brain-derived neurotrophic factor, neurotrophin- (NT-) 3, IGF-I, IGF-II, and glial cell-derived neurotrophic factors have also been examined as therapeutic agents [5, 32].

However, this study have several limitations. First, although the NGF agonistic activity and anti-inflammatory effect of DA-9801 was proven to contribute to the prevention of peripheral nerve damage, more detailed mechanism and identification of the active components of Dioscorea species are warranted for the effective use of DA-9801 in DPN management. Second, dose-response effect of DA-9801 on diverse parameters in DPN assessment should be investigated. Third, NGF pathway, in the pathophysiologic treatment of DPN, is considered to lead nerve regeneration as well as neuronal survival. However, an examination of their differences was not performed in the present study. Further investigation on the effects of DA-9801 for the treatment of DPN should be conducted. 

In summary, the results of this study suggest the potential of DA-9801 for the prevention of degeneration or induction of regeneration of peripheral nerves in diabetic rats. Furthermore, this therapeutic potential is greater in DA-9801 than in ALA. The effects of DA-9801 are believed to include enhancement of neurotrophic activity and anti-inflammatory response, which are commonly involved in the pathogenesis of DPN. The improvement of NGF and decreased levels of TNF-*α* and IL-6 may reduce the deterioration of the peripheral nerves by chronic hyperglycemia. However, DA-9801 has not completely prevented the hyperglycemia-induced metabolic disturbance in the peripheral nerves. Therefore, multiple steps involved in the pathogenesis of DPN need further assessment to attain more effective pathogenic correction or complete prevention of DPN progression.

## Abbreviations

ALA: Alpha lipoic acid
DM: Diabetes mellitus
DPN: Diabetic peripheral neuropathy
NGF: Nerve growth factor
IENF: Intraepidermal nerve fiber
IL: Interleukin
TNF: Tumor necrosis factor.
